# Oral health, stress and barriers accessing dental health care among war-affected Ukrainian refugees in Germany

**DOI:** 10.1186/s12903-023-03513-x

**Published:** 2023-10-27

**Authors:** Maksym Ponomarenko, Andrea Kaifie

**Affiliations:** Institute for Occupational, Social, and Environmental Medicine, Medical Faculty, RWTH Aachen University, Pauwelsstrasse 30, 72074 Aachen, Germany

**Keywords:** Unmet needs, Stress, Anxiety, Asylum seeker, Barriers accessing dentist

## Abstract

**Background:**

After Russian invasion many Ukrainians fled to European countries including Germany. In this context, the German health care system faced challenges delivering dental care to a displaced population. Recently surfaced obstacles as well as different cultural and medical traits need to be considered in order to deliver appropriate medical care. The aim of this study was to evaluate oral health and hygiene of Ukrainian refugees, identify barriers accessing dental health care and explore the relation to their mental health state.

**Methods:**

This cross-sectional study was conducted using a self-assessment questionnaire, distributed via non-probability snowball sampling method among war-affected Ukrainians, who fled to Germany. The online form was distributed via web-based platforms, the printed version was hand-delivered across diverse local venues. Chi-Square Tests, T-Tests and Mann-Whitney-U Tests were performed. Analysis of variance and Spearman correlation coefficient analysis were also conducted.

**Results:**

From 819 completed questionnaires, 724 questionnaires were included in the analysis with 78 males (10.8%) and 640 females (88.6%) and a mean age of 37.5 years (SD = 10.5). The majority of participants rated their state of teeth (77%) and gums (81%) as average or better. The main problems, caused by state of their teeth, were: “Have avoided smiling because of teeth” (23.6%) or “Felt embarrassed due to appearance of teeth” (22.2%). The most frequent limiting factors to access dental care were finances (82.6%), language (82.2%) and complicated health care system (74.1%). 45.8% of the participants scored 10 or more in the Patient Health Questionnaire and 37.4% in the Generalized Anxiety Disorder 7-item scale, respectively. These participants were more likely to report pain, poor state of teeth and gums and to fail a dental consultation. Overall, 59.6% participants reported not consulting a dentist, when needed. Failed consultations were associated with a poorer reported state of teeth and gums.

**Conclusions:**

Ukrainian refugees reported barriers accessing dental health care in Germany. It is important to improve oral health literacy and dental services for displaced people and provide help and guidance in seeking dental care.

**Supplementary Information:**

The online version contains supplementary material available at 10.1186/s12903-023-03513-x.

## Background

As a result of various conflicts, wars and violence led to more than 40 million refugees and asylum seekers worldwide at the end of 2022 according to the United Nations Refugee Agency [1]. The ongoing war in Ukraine forced 6.3 million Ukrainian refugees to flee their homes seeking safety as of June 2023 [1]. Germany alone welcomed over 1 million Ukrainian refugees [2]. However, this also created new challenges for the health care system and in particular, for dental care.

Dental care is important for wellbeing and overall health [3]. Impaired oral health is associated with heart disease, mental health problems as well as respiratory infections [4–7]. Therefore, it is essential to maintain adequate dental care for all people, including refugees and asylum seekers. While escaping war and violence they often had only restricted access to basic health care services in their home country or in a country of temporary stay [8]. The level of oral health, awareness of dental hygiene practices and overall level of healthcare knowledge varies across different countries [9]. Therefore, dentists in host countries maybe confronted with unique challenges while treating patients from other regions.

Despite successful efforts to welcome and integrate displaced populations, refugees often face various barriers and limitations accessing dental health care [10–16]. Additionally, their priorities throughout the resettlement phase are mainly the integration into the host nation, learning a new language, finding sources of income, and establishing a normal level of life. As a result, dental care and hygiene frequently play a rather subordinate role to other urgent needs. This impacts oral health behavior and results in high caries experiences, untreated teeth and additional complications [17–19]. The understanding of actual needs and barriers could help to identify successful strategies of providing appropriate dental care.

War refugees from Ukraine were able to enter Germany without a visa or residence permit and were allowed to stay for 90 days (until 31.08.2022) without registration [20]. However, if they wanted to stay for a longer period of time or receive social assistance, they needed to apply for a humanitarian residence permit from the immigration office. Before this, Ukrainian refugees were entitled to benefit from the Asylum Seekers Beneficts Act (AsylbLG) meaning their access to healthcare was restricted and included only treatment of acute pain and illnesses[21].

Previous studies examining oral health of Ukrainian refugees immigrating to Germany after the beginning of the 2022 war are limited. The aim of this study is to determine the status of oral health among newly arrived refugees, explore their oral health practices, identify barriers and limitations accessing dental care in Germany and investigate their mental state in relation to oral health problems.

## Methods

### Study design, participants and data collection

This cross-sectional study took place in North Rhine-Westphalia, Germany between September and December 2022 and included people with Ukrainian citizenship, which flew their home country because of the war. All participants required to be at least 14 years old. Ukrainians, who traveled to Germany before the war, and were not able to get back to Ukraine were also included.

In order to reach more participants, two versions of the questionnaire were distributed: a paper-based and a web-based version, distributed via non-probability snowball sampling method. The web-based questionnaire was developed with SoSci Survey (SoSci Survey GmbH, Munich, Germany) and was available online at www.soscisurvey.de [22]. Information about the study with a link to the survey was shared on internet resources, related to Ukrainian refugees in different German cities. The paper-based version was distributed personally through local community centers, local organizations and other places of interest, frequently visited by Ukrainians. The investigator, who is fluent in Ukrainian, shared information about the study and offered voluntary participation during the visits. Ethical approval for the study was obtained from the local Ethics Committee of the RWTH Aachen University (EK22-292, 15 September 2022). At the beginning of survey all participants were informed about aim of the study and their anonymity. Participation in this study was voluntary.

### Questionnaire development

Our questionnaire consisted of 35 items, including sub-questions, and covered the following general topics:

1. Filter questions.
2. General demographic information.
3. Oral health status and practices.
4. Dental care access.
5. Unmet needs for dental health services.
6. Stress and anxiety measurements.

In this study, mainly already validated tools were used in order to produce standardized data and compare it to other studies [23]. Information on oral health status and practices was based on WHO’s (World health Organization) manual and self-assessment survey “Oral Health Surveys – Basic Methods”[24] in line with different studies regarding oral health [16, 19, 25]. For stress and anxiety measurement, the modules from the Patient Health Questionnaire Somatic, Anxiety, Depressive Symptoms (PHQ-SADS) were used: the 9-items containing Patient Health Questionnaire (PHQ-9) and the Generalized Anxiety Disorder 7-item scale (GAD-7). These scales are well-validated and widely used instruments for the screening and monitoring of depression and anxiety with high sensitivity and specificity [26–30]. A cut-off score of 10 was chosen implying at least moderate depression or anxiety levels. The oral health and barriers part of the questionnaire was translated to the main languages spoken in Ukraine, Ukrainian and Russian. The bilingual investigator checked for cross-language equivalence [31]. PHQ-9 und GAD-7 scales were already available and validated in both languages [32]. The English version of the complete questionnaire can be found in the supplemental section (see **Additional file 1**).

After finalizing, the questionnaire was pilot-tested by a group of 10 Ukrainian participants. Despite of minor grammatical improvements, they reviewed the survey as clear and easy to understand.

At the beginning of the survey, participants had to answer two filter questions: “Are you a Ukrainian citizen?” and “Did you flee to Germany since February 2022?” If both answers were answered with “Yes”, the participant could proceed with the questionnaire.

Demographics included questions about gender, age, marital status, education, language skills, place of residence in Ukraine and in Germany, health insurance, and accompanying family or friends.

The part concerning oral health status and practices included questions about state of teeth and gums, pain experience, oral hygiene practice, visiting a dentist in Ukraine and in Germany, difficulties in everyday life because of teeth, nourishment, smoking and drinking alcohol.

The dental care access part contained ratings of barriers in form of a 6-point Likert scale. After literature research the main barriers, defined in previous qualitative studies [13, 15, 33], were chosen and included for quantitative exploring. These were: language barriers, financial barriers, transport barriers, availability in home region, difficulties understanding dental health care system, difficulties finding a dentist, dental anxiety, trust issues with the dentist, cultural and religious beliefs, social barriers. Moreover, in the unmet needs part, the participants were asked if they failed a consultation, meaning if there was a time when a participant wanted to consult a dentist but did not. Subsequent to this question, the participants could state, which barriers led to this failed consultation.

### Statistical analysis

The primary survey data was analyzed using Statistical Analysis System Software (SAS Studio Release 3.8 Enterprise Edition, SAS Institute Inc., Cary, NC, USA). Descriptive statistics were used for all participants in order to describe the most important trends. As a result, frequency analysis was performed for all categorical variables. Medians, interquartile ranges and standard deviations were calculated for continuous variables. Chi-Square Tests, T-Tests and Mann-Whitney-U Tests were performed. Analysis of variance was performed to describe relation between categorical and continuous variables. Spearman correlation coefficient was used to calculate association between ordinal data. Likert Scales and some other variables were also dichotomized in order to run Chi-Square Tests. Some categorical variables were simplified for statistical analysis. The level of significance was set at 5%.

## Results

### Sociodemographics

Overall n = 819 participants participated in the survey. Altogether, n = 724 questionnaires were included in the analysis with n = 78 males (10.8%) and n = 640 females (88.6%), as described in Table 1. The mean age of the participants was 37.5 years (SD = 10.5). Over half of the study participants were married (51.9%; n = 375;), followed by singles (19.4%; n = 140) and divorced (15.9%; n = 115). In terms of education, most of the participants (72.6%; n = 523) had completed higher education and approximately 17% (n = 121) vocational or technical education. Half of the participants rated their level of English as average or better, but only 11.6% (n = 81) felt the same way about their German. Most of the refugees came from Eastern Ukraine (38%; n = 274) and large cities (66.3%; n = 478). Additionally, the majority (82.9%; n = 599) arrived in Germany with their families. More than 78% (n = 568) of the participants have been staying in Germany for at least 4 months. Less than 9% (n = 58) of the participants lacked health insurance in Germany, all other refugees were already insured.

**Table 1:** Sociodemographic information about the participants

Sex (n (%))		Age (mean (SD))	
Female	640 (88.6)	37.5 (10.5)	
Male	78 (10.8)		
Diverse	4 (0.6)		
**Marriage status (n (%))**		**Education (n (%))**	
Married	375 (51.9)	Higher Education	523 (72.6)
Single	140 (19.4)	Vocational or technical education	121 (16.8)
Divorced	115 (15.9)	General secondary education	59 (8.2)
In a relationship	77 (10.6)	Other	17 (2.4)
Widowed	16 (2.2)		
**Level of English (n (%))**		**Level of German (n (%))**	
Very poor	164 (24)	Very poor	412 (58.9)
Poor	174 (25.5)	Poor	206 (29.5)
Average	213 (31.2)	Average	58 (8.3)
Good	88 (12.9)	Good	14 (2)
Very good	44 (6.4)	Very good	9 (1.3)
**Home region in Ukraine (n (%))**		**Size of home city in Ukraine (n (%))**	
Eastern Ukraine	274 (38)	Large city	478 (66.3)
Central Ukraine	257 (35.6)	Medium city	131 (18.2)
Southern Ukraine	134 (18.6)	Small city	82 (11.4)
Western Ukraine	56 (7.8)	Village or urban-type village	30 (4.1)
**Came alone or with family? (n (%))**		**Size of city in Germany (n (%))**	
With family members	599 (82.9)	Large city	238 (33.2)
Alone	87 (12)	Medium city	235 (32.7)
With friends / acquaintances	29 (4)	Small city	141 (19.6)
With other people	8 (1.1)	Village or urban-type village	104 (14.5)
**Time in Germany (n (%))**		**Insurance (n (%))**	
1–2 months	50 (6.9)	No	58 (8.2)
3–4 months	85 (11.8)	Yes	646 (91.8)
4–6 months	345 (47.8)		
Less than 1 month	19 (2.6)		
More than 6 months	223 (30.9)		

### Oral health

The overall reported oral health status was at a good level among the participants. Most of them described the state of their teeth at least average (77%; n = 542). The state of gums was slightly better, only 9.1% (n = 76) reported poor or very poor gums. Moreover, the participants showed very good oral hygiene practices (Table 2). Most of the participants cleaned their teeth once, twice or more times a day (49.9%; n = 357 and 44.4%; n = 318 respectively) with toothbrush (98.3%; n = 318) and toothpaste (99.9%; n = 712). Although around 45% (n = 305) of the participants used tooth paste with fluoride, 41% (n = 281) did not know if their toothpaste contained fluoride and 14% (n = 97) did not use fluoride tooth paste. The most frequent additional tooth cleaning products were floss (40%; n = 284) and toothpick (22.3%; n = 160).

**Table 2:** Oral Health

State of teeth (n (%))		State of gums (n (%))		
Very poor	44 (6.2)	Very poor		13 (1.9)
Poor	118 (16.8)	Poor		50 (7.2)
Average	282 (40.1)	Average		241 (34.7)
Good	178 (25.3)	Good		247 (35.6)
Very good	65 (9.2)	Very good		88 (12.7)
Excellent	17 (2.4)	Excellent		55 (7.9)
**Frequency of cleaning teeth (n (%))**		**Using toothbrush (n (%))**		
Twice or more a day	318 (44.4)	Yes	705 (98.3)	
Once a day	357 (49.9)	No	12 (1.7)	
2–6 times a week	31 (4.3)			
Once a week or more rarely	10 (1.4)			
**Consuming sweets or candies (n (%))**		**Smoking cigarettes (n (%))**		
At least every day	132 (19.2)	Every day	104 (15.2)	
Several times a week	252 (36.8)	Several times a week	15 (2.2)	
Once a week	93 (13.6)	Once a week	2 (0.3)	
Several times a month	139 (20.3)	Several times a month or more rarely	51 (7.5)	
Seldom/never	69 (10.1)	Never	511 (74.8)	
**Reason for last dental visit in Germany (n (%))**		**Frequency of visiting dentist in Ukraine (n (%))**		
Pain or trouble with teeth, gums or mouth	169 (49.7)	Twice a year or more	304 (44.4)	
Treatment / Follow up treatment	107 (31.5)	Once a year	217 (31.7)	
Consultation / advise	33 (9.7)	Less than once a year	141 (20.6)	
Routine check-up	31 (9.1)	I do not know	19 (2.8)	
		Never received dental care	3 (0.5)	
**Reason for last dental visit in Ukraine (n (%))**		**Pain during last 12 month, caused by teeth or mouth? (n (%))**		
Pain or trouble with teeth, gums or mouth	118 (22.1)	Yes	558 (78.7)	
Treatment / Follow up treatment	253 (47.4)	No	147 (20.7)	
Consultation / advise	53 (9.9)	I do not know	4 (0.6)	
Routine check-up	110 (20.6)			
**Failed a consultation (n (%))**		Reasons for failed consultation (n (%))*		
Yes	390 (59.6)	Language barriers	260 (66.5)	
No	264 (40.4)	Financial barriers	246 (62.9)	
**Started treatment in Ukraine (n; %)**		Problems with finding a dentist	219 (56)	
Yes	252 (41.7)	Complicated health care system	162 (41.4)	
No	353 (58.3)	Dental anxiety	76 (19.4)	
**Continued treatment in Germany (n; %)**		Trust issues	68 (17.4)	
Yes	79 (27.3)	Social barriers	42 (10.7)	
No	146 (50.3)	Transport barriers	41 (10.5)	
Did not search for a dentist	65 (22.4)	Availability in region	31 (7.9)	
		Cultural and religious beliefs	0 (0)	
		Other	27 (6.9)	
**Variable**	**Groups**	**Value**	**p**	
State of teeth (mean (SD))	With higher education	3.4 (1.0)	< .0001	
	Other	2.8 (1.2)		
State of gums (mean (SD))	With higher education	3.8 (1.1)	.0010	
	Other	3.5 (1.1)		
Last time visited dentist for check-up (n (%))	With higher education	120 (24.1)	.0002	
	Other	21 (11.2)		

* - Multiple choice was available

Regarding food habits, fresh fruits were popular. More than half of participants (n = 346; 50.5%) reported to consume them every day or more often. 36.2% (n = 248) ate fresh fruits several times a week and only 3.2% (n = 22) ate them seldom or never ate them. Sweets or candies were mostly consumed several times a week. When the participants were asked if they smoke cigarettes, the majority reported (74.8%; n = 511) to have never smoked.

A significant predictor for oral health was education. Participants with a higher education reported a better state of teeth (mean 3.4 (SD 1.0)) and gums (mean 3.8 (SD 1.1)) compared to other participants (mean 2.8 (SD 1.2) and mean 3.5 (SD 1.1)), respectively. In addition, participants with higher education more often visited a dentist for a dental check-up routine (69.3%; n = 377).

Most of the participants (n = 521; 76.2%) were visiting a dentist at least once a year. However, around 79% (n = 558) have suffered from pain, caused by teeth or mouth, within the last 12 months. Pain was also the main reason for visiting a dentist in Germany (49.7%; n = 169). The main reason for visiting a dentist in Ukraine was treatment / follow-up treatment (47.4%; n = 253), followed by pain / trouble with mouth (22.1%; n = 118). Routine check-ups were the less frequent reason for visiting a dentist in Germany (9.1%; n = 31). In Ukraine 20.6% of participants (n = 110) visited a dentist for a check-up routine.

**Fig. 1:**
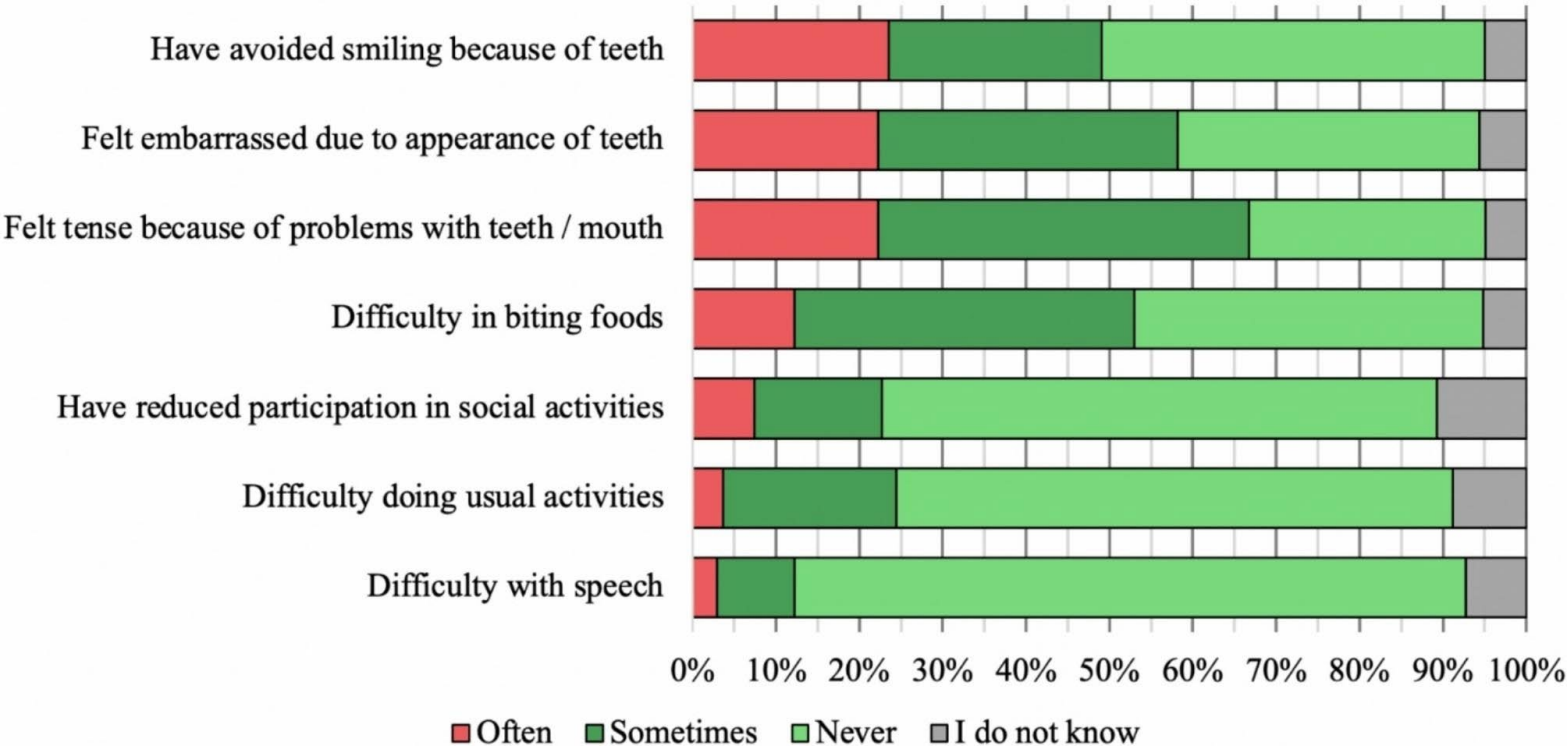
Reported problems because of the status of the teeth

When participants were asked to rate different problems, caused by the state of their teeth, the main reported problems were: “Have avoided smiling because of teeth” (23.6%), “Felt embarrassed due to appearance of teeth” (22.2%), “Felt tense because of problems with teeth/mouth” (22.2%), as depicted in Fig. 1.

### Limiting factors

**Fig. 2:**
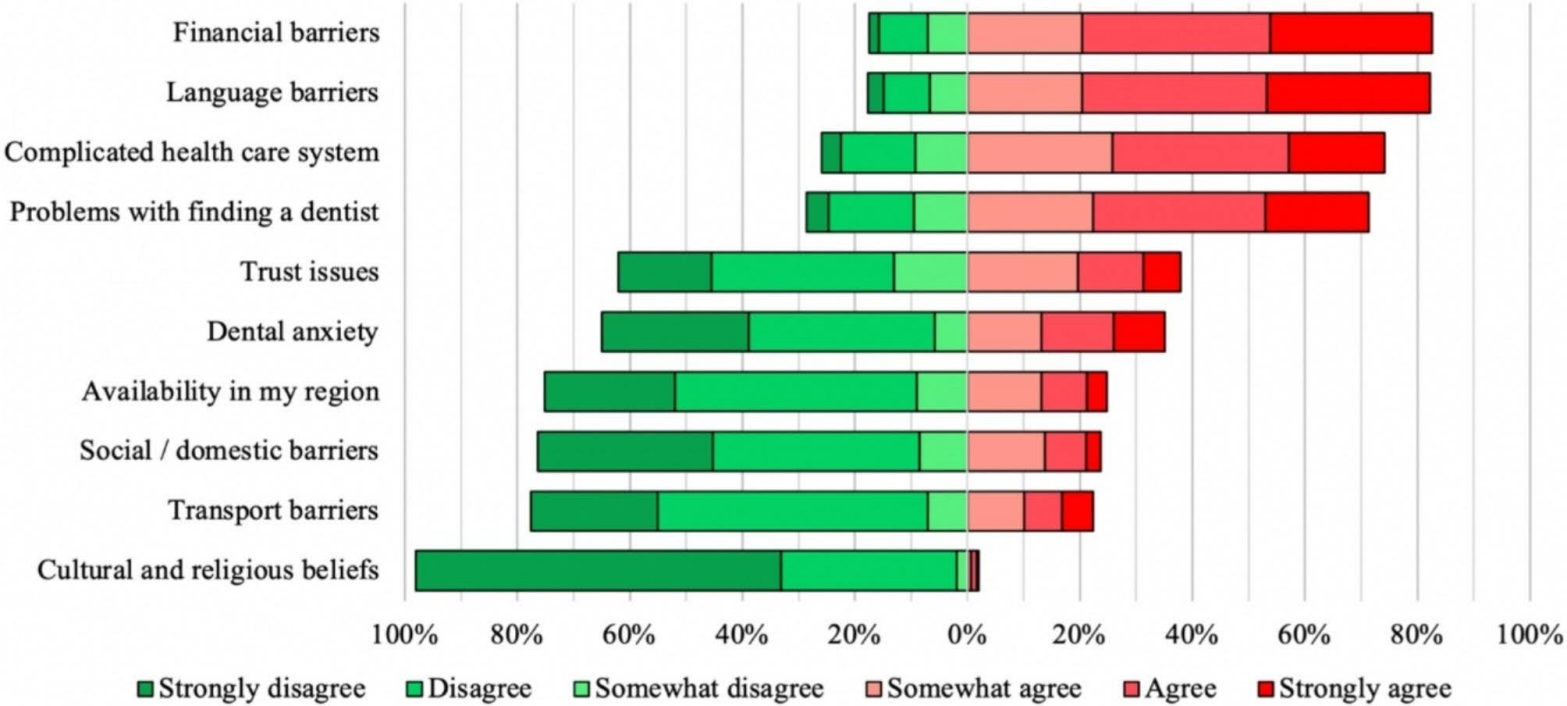
Limiting factors accessing dental health care

Figure 2 demonstrates how much different factors in opinion of participants limit their access to a dentist in Germany.

Financial barriers were the most reported limiting factor to access dental care, stated by 82.6% (n = 540) of the participants (at least somewhat agree). Language barriers were reported by 82.2% (n = 536) of the participants. 74.1% (n = 484) at least somewhat agreed, that the complicated health care system was a limiting factor, as well as problems with finding a dentist (n = 466; 71.4%).

There was no statistically significant difference in limiting factors between males and females, except for trust issues (see Table 3). Females were more likely to report trust issues (39.6%) compared to males (21.9%). Age was a significant predictor for language barriers, trust issues, dental anxiety and social/domestic barriers. Participants, who reported language barriers were older (mean age 37.8 years (SD 10.6)) compared to those without reporting language barriers (mean age 35.3 years (SD 10.5)). Younger participants (mean age 35.1 years (SD 9.0)) reported trust issues more often than older participants (mean age 38.8 years (SD 11.4)). Trust issues were significantly associated with the size of city in Germany.

**Table 3:** Limiting factors and failed consultations

	Limiting factors							Failed a consultation
	Financial barriers	Language barriers	Complicated health care system	Problems with finding a dentist	Trust issues	Dental anxiety	Social / domestic barriers	
**Gender (n (%))**								
Males	52 (80)	53 (81.5)	53 (81.5)	47 (72.3)	14 (21.9)*	19 (29.2)	11 (17.2)	34 (53.1)
Females	482 (82.7)	478 (82.3)	428 (73.5)	414 (71.1)	230 (39.6)*	207 (35.6)	139 (24.1)	352 (60.3)
**Age (mean (SD))**								
Experienced this barrier	37.6 (10.7)	37.8 (10.6)*	37.3 (10.4)	37.3 (10.7)	35.1 (9.0)**	36.2 (10.6)*	33.9 (8.3)**	37.02 (9.64)
Did not experience this barrier	36.9 (10.6)	35.3 (10.5)*	37.8 (11.4)	37.7 (10.7)	38.8 (11.4)**	38.1 (10.7)*	38.4 (11.0)**	37.90 (11.78)
**Size of the city in Germany (n (%))**								
Large or medium	450 (81.8)	445 (81.2)	411 (74.9)	399 (72.4)	218 (39.8)*	185 (33.6%)	128 (23.5)	
Small or village	89 (86.4)	90 (87.4)	73 (70.9)	66 (65.4)	28 (27.5)*	43 (42.6%)	25 (24.5)	
**Arrived alone or with family (n (%))**								
Alone	101 (88.6)	89 (78.8)	79 (69.9)	86 (75.4)	39 (34.8)	38 (33.3)	14 (12.6)*	55 (48.3)*
With family	439 (81.3)	447 (82.9)	405 (75.0)	380 (70.5)	208 (38.6)	191 (35.4)	139 (25.9)*	335 (62.0)*

** - p ≤ .0001; * - p < .05

### 3.4. Unmet dental needs

When asked if there if there was a time in Germany when they needed to consult a dentist but did not, 59.6% (n = 390) answered with “yes” (see Table 2). They were subsequently asked for the reasons of the failed consultation. There was a statistically significant difference between people, who came with family to Germany, with regard to the frequency of failed consultations compared to participants who did not came together with their family (see Table 3). Participants with family were more likely to not visit a dentist (n = 335; 62.0%) when they needed one compared to participants who came to Germany alone (n = 55; 48.3%). There was no difference concerning other demographic characteristics (gender, age, education) (see **Additional File 2**). Interestingly, participants, who reported an unsuccessful consultation were more likely to report a poor or a very poor state of teeth (n = 111; 74.5%) and gums (n = 44; 75.9%), compared to other participants (n = 271; 54.9% and n = 337; 58.1% respectively).

41.7% (n = 252) of the participants have started a dental treatment in Ukraine (Table 2) that needed to be continued in Germany. However, only 27.3% (n = 79) continued the treatment, while 22.4% (n = 65) did not search for a dentist and 50.3% (n = 146) were not able to continue the treatment in Germany.

### Stress and anxiety

The mean PHQ-9 score to screen for depression was 9.9 (SD 6.01) and the mean GAD-7 score to detect anxiety was 8.5 (SD 5.26). 45.8% of the participants (n = 292) scored 10 or more in PHQ-9 and 37.4% (n = 237) scored 10 or more in GAD-7 indicating at least moderate symptoms. There was a significant association between gender and PHQ-9 with females reporting higher mean scores compared to males (see Table 4). Also, for GAD-7 females reported higher mean scores with 8.83 (SD 5.27) compared to males with a mean score of 5.59 (SD 3.99).

**Table 4:** Mean PHQ-9 and GAD-7 scores for males and females

	PHQ-9 (mean score (SD))	GAD-7 (mean score (SD))
Males	6.48 (4.45) ***	5.59 (3.99) ***
Females	10.24 (6.05) ***	8.83 (5.27) ***
*** - p ≤ .0001		

There was a statistically significant relation between stress or anxiety and oral health (see Table 5). Participants with PHQ-9 score ≥ 10 were more likely to report pain, caused by teeth or mouth (84.1%; n = 243) than participants with a PHQ-9 score < 10 (76.3%; n = 260) The same relation was observed for GAD-7 (86.9%; n = 205 and 75.5%; n = 295 respectively). Participants with a PHQ-9 score ≥ 10 reported more often a poor or a very poor state of teeth (27.4%; n = 78) and gums (13.8%; n = 39) compared to participants with a PHQ-9 score < 10 (19.4%; n = 66 and 5.3%; n = 18 respectively). Participants reporting a GAD-7 score ≥ 10 rated more often that their state of teeth (28.0%; n = 65) and gums (14.4%; n = 33) was poor / very poor compared to participants with a GAD 7-score < 10 (20.0%; n = 78 and 5.9%; n = 23 respectively). Overall, high PHQ-9 and GAD-7 scores were associated with a reported feeling of being embarrassed due to the appearance of teeth and a reduced participation in social activities. Interestingly, participants with PHQ-9 score ≥ 10 (64.7%; n = 189) or GAD-7 ≥ 10 (70.5%; n = 167) were less likely to consult a dentist, when needed, and reported failed consultations more frequent compared to participants who scored less than 10 in the PHQ-9 (54.9%; n = 189) and GAD-7 (52.8%; n = 209) scales.

**Table 5:** Relation between stress or anxiety and oral health

	PHQ-9 ≥ 10	GAD-7 ≥ 10
Pain, caused by teeth or mouth (n (%))	243 (84.1)*	205 (86.9)**
State of teeth is poor / very poor (n (%))	78 (27.4)*	65 (28.0)*
State of gums is poor / very poor (n (%))	39 (13.8)**	33 (14.4)**
Feeling embarrassed due to appearance of teeth (n (%))	185 (66.8)*	152 (68.5)*
Reduced participation in social activities (n (%))	82 (31.4)*	72 (34.3)**
Failed consultations (n (%))	189 (64.7)*	167 (70.5)***

*** - p ≤ .0001; ** - p < .001; * - p < .05

## Discussion

In this study a high prevalence of limitations accessing dental health care and unmet needs in line with high stress and anxiety levels was observed. To the best of our knowledge, this is the first study, that covers these topics in the population of Ukrainian refugees in Germany.

Considering the sociodemographic data in this study, the population of Ukrainian refugees showed important differences compared to previous studies on refugees. Participants were predominantly middle-aged women with a high level of education. This differs from previous studies, where most of the participants were younger males with a lower level of education [10, 16, 19, 33, 34]. Therefore, specific dental care needs differ from previous studies. A low level of German as well as an average level of English could play a significant role in terms of barriers accessing dentist, which will be further discussed.

Previous studies suggest, that oral health literacy is associated with oral health [35, 36]. The reported good state of teeth and gums, and the reported good oral hygiene practices as well as the frequent visit of a dentist in Ukraine allows the assumption that the level of oral health literacy was high among the participants. However, high uncertainty regarding the presence of fluoride in toothpaste and infrequent check-ups left room for improvement. Life circumstances, such as a current war in the home country and adapting to a new country may be an explanation among this particular group of refugees [37]. According to a recent study among the population in Germany [38] and the German Oral Health Study 5 (Deutsche Mundgesundheitsstudie V) [39], at least 60% of participants visited a dentist for regular check-ups, which is considerably higher than the reported 21% of check-ups in refugee’s home country and demonstrates a need to further improve preventive dentistry literacy among refugees, also in their home countries. In line with this study, a high level of concerns regarding aesthetic and appearance of teeth was observed.

Participants reported a high level of barriers accessing dental care, such as finances, language and a complicated health care system. Although, most participants already had active health insurance in Germany, only basic dental treatment is covered by the health insurance. However, barriers in dental care needs of refugees exceed financial strains and additional factors need to be considered, as well. Providing clearer information about the dental health system and costs in the refugees’ native language could help to overcome this fear and increase the uptake. Participants, who reported unsuccessful consultations were more likely to report a poor or a very poor state of teeth and gums. Various factors, that are associated with missed or cancelled dental appointments, such as self-paying for dental care [40], high caries experience, negative beliefs of dentists and others [41]. These factors were reflected in barriers and limitations, experienced by Ukrainian refugees. Therefore, missing dental consultations would likely have a negative impact on dental health. Conversely, a bad state of teeth and gums could also lead to dental anxiety and unattendance [42]. This underscores the importance of encouraging dental services and making it accessible among this population.

With regard to mental health, the mean PHQ-9 and GAD-7 scores of participants were significantly higher among the study population compared to the German population, indicating a high mental burden [43]. According to a recent study in Germany, only 31.1% exceeded the cutoff score for depressive symptoms compared to 45.8% of the Ukrainian participants, and 21.2% exceeded the cutoff score for anxiety compared to 37.4% in this study population [44]. As expected, stress and anxiety levels were higher among the Ukrainian refugees and especially among females. This high mental burden may explain the frequent failed dental consultations. Moreover, there was a statistically significant relation between stress or anxiety and oral health. Participants who exceeded the cut-off scores for GAD-7 and PHQ-7 reported more frequent pain, felt embarrassed or rated their state of teeth and gums as bad. These findings indicate a link between mental health and oral health, and are presenting the influence of mental health on self-perception. Therefore, it is important for dentists to understand the unique needs of Ukrainian refugees, identify specific problems and possible solutions. Dentists should improve their knowledge on post-traumatic syndromes, behavioral sciences or psychology in general and in particular regarding war-affected people.

Finally, it is important to mention the limitations of this study. Due to limited resources, the oral health of the participants was assessed with a self-assessment questionnaire and not by clinical examination. A low accuracy of self-reported questionnaires might be considered [45], although other authors stated that self-reported oral health is associated with normative indices of oral health [46]. Further, non-probability sampling methods were used. This could have led to a selection bias and reducing the likelihood of a representative sample of Ukrainian refugees in Germany. It is possible, that refugees, who encountered more limitations while accessing dental health care were more interested in participating in this study. Finally, the cross-sectional design of this study is not allowing to establish causalities. Despite these limitations, our study represents the first known examination of oral health among Ukrainian refugees in Germany, and thus provides an important contribution to the literature.

There are still significant knowledge gaps about specific needs of Ukrainian refugees. The ongoing war in Ukraine resulted in a relatively new displaced population with different cultural and medical needs, compared to previous displaced people. Further research is required in order to assess these needs and improve access to optimal dental services.

## Conclusions

The present study shows a high prevalence of barriers and limitations accessing dental health care among Ukrainian refugees in Germany. Together with high stress and anxiety levels these barriers and limitations could lead to unmet dental needs and subsequent worsening of oral health. Despite of some well-known barriers, there are differences compared to other displaced populations. Therefore, it is important to improve dental services for displaced people, consider their unique needs, provide financial and informational guidance, and prepare health workers to face novel challenges in dental care services.

### Electronic supplementary material

Below is the link to the electronic supplementary material.

Supplementary Material 1

Supplementary Material 2

## Data Availability

The dataset(s) supporting the conclusions of this article can be made available upon request from the corresponding author.
