# Supplementary material for: Oral health, stress and barriers accessing dental health care among war-affected Ukrainian refugees in Germany
Source: BMC Oral Health. 2023 Oct 27;23:804. doi: 10.1186/s12903-023-03513-x (PMC10612176; doi:10.1186/s12903-023-03513-x)
Supplement: Supplementary file 1 — Supplementary Material 1 [file 12903_2023_3513_MOESM1_ESM.doc]

# General information about the study

**Aim of the study:** In this study you are going to anonymously answer questions about the dental care in Germany. The aim of the study is to research the accessibility of dental care for Ukrainian refugees, which barriers and difficulties result.

**Participation requirements:** You are at least 14 years old Ukrainian citizen and fled to Germany since February 2022

**Duration:** This questionnaire will take approximately 10 minutes to complete.

**Anonymity:** All information you provide here will be saved and processed anonymously. This means that no information can be traced back to you personally.

**Contact:** If you have any questions about this study, you will find the contact details of the study directors at the end of survey.

**Your participation in this study is voluntary. There are no disadvantages for you if you do not participate or discontinue your participation at any time during this study!**

By completing this questionnaire, you confirm that you have read and understood the information given above and that you consent to the anonymous processing of your data.

1. Are you Ukrainian citizen?
   - Yes
   - No
   - I don‘t know
2. Did you flee to Germany since February 2022?
   - Yes
   - No
   - I don‘t know

**Demographic information**

1. Sex:
   - Male
   - Female
   - Divers
2. How old are you? __________________________________
3. Marriage status:
   - Single
   - Married
   - In a relationship
   - Divorced
   - Widowed
4. What is the highest level of education you have completed?
   - Currently pupil at school
   - General secondary education
   - Vocational and technical education
   - Higher Education
   - Other
5. Language level:

|  | **Very good** | **Good** | **Average** | **Poor** | **Very poor** |
| --- | --- | --- | --- | --- | --- |
| a) **English** | 🞏 | 🞏 | 🞏 | 🞏 | 🞏 |
| b) **German** | 🞏 | 🞏 | 🞏 | 🞏 | 🞏 |

1. Where did you live permanently in Ukraine?


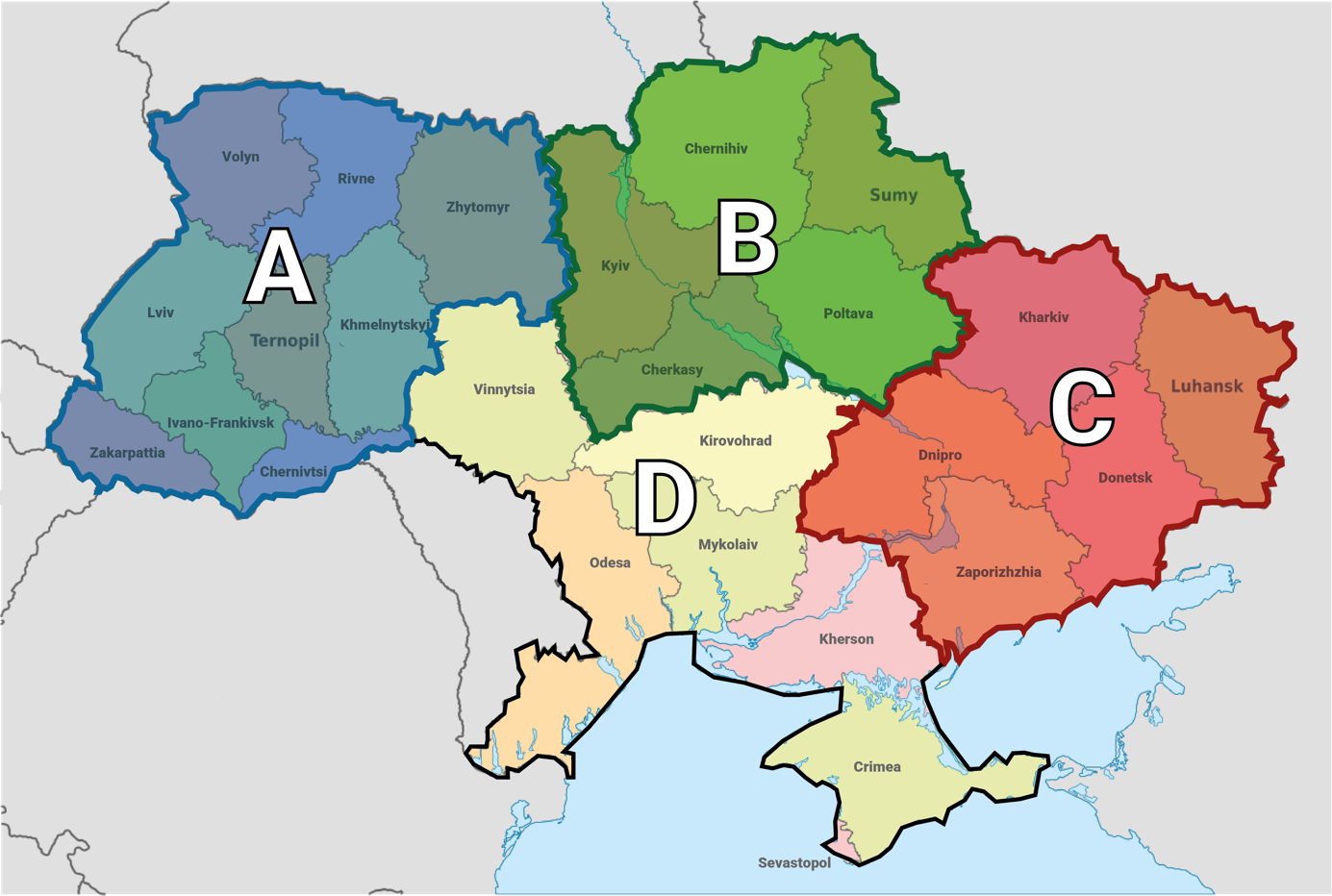


- - A - Western Ukraine
  - B - Central Ukraine
  - C - Eastern Ukraine
  - D - Southern Ukraine

1. Which of these best describes place where you lived in Ukraine?
   - Large city (population equal or more than 500 000)
   - Medium sized city (population 100 000 - 500 000)
   - Small city (less than 100 000)
   - Village or urban-type village
2. Which of these best describes place where you currently staying in Germany?
   - Large city (population equal or more than 500 000)
   - Medium sized city (population 100 000 - 500 000)
   - Small city (less than 100 000)
   - Village or urban-type village
3. How long are you already in Germany?
   - < 1 month
   - 1-2 months
   - 3-4 months
   - 4-6 months
   - > 6 months
4. Do you have German health insurance?
   - Yes
   - No
   - I don’t know
5. Did you arrive in Germany alone or together with family members or with friends / acquaintances? (Multiple answers possible)
   - Alone
   - With family members
   - With friends / acquaintances
   - With other people

**Oral health status and practices**

1. How many natural teeth do you have?
   - No natural teeth
   - 1 – 9 natural teeth
   - 10 – 19 natural teeth
   - 20 teeth or more
2. During the past 12 months, did your teeth or mouth cause any pain or discomfort?
   - Yes
   - No
   - Don’t know
   - No answer
3. Do you have any removable dentures?

|  | **Yes** | **No** |
| --- | --- | --- |
| A partial denture? | 🞏 | 🞏 |
| A full upper denture? | 🞏 | 🞏 |
| A full lower denture? | 🞏 | 🞏 |

1. How would you describe the state of your teeth and gums? Is it “excellent”, “very good”, “good”, “average”, “poor”, or “very poor”?

|  | **Excellent** | **Very good** | **Good** | **Average** | **Poor** | **Very poor** |
| --- | --- | --- | --- | --- | --- | --- |
| a) **Teeth** | 🞏 | 🞏 | 🞏 | 🞏 | 🞏 | 🞏 |
| b) **Gums** | 🞏 | 🞏 | 🞏 | 🞏 | 🞏 | 🞏 |

1. How often do you clean your teeth?
   - Never
   - Once a month
   - 2–3 times a month
   - Once a week
   - 2–6 times a week
   - Once a day
   - Twice or more a day
2. Do you use any of the following to clean your teeth?

|  | **Yes** | **No** |
| --- | --- | --- |
| Toothbrush | 🞏 | 🞏 |
| Toothpick | 🞏 | 🞏 |
| Thread (dental floss) | 🞏 | 🞏 |
| Charcoal | 🞏 | 🞏 |
| Chewstick/miswak | 🞏 | 🞏 |
| Other | 🞏 | 🞏 |
| Please specify_______________________________________________________ | | |

- 1. Do you use toothpaste to clean your teeth?
     - Yes
     - No
     - Don’t know
  2. If you answered previous question with “Yes”: Do you use a toothpaste that contains fluoride?
     - Yes
     - No
     - Don’t know

1. How long is it since you last saw a dentist?
   - Less than 6 months ago
   - 6–12 months ago
   - More than 1 but less than 2 years ago
   - 2 or more years ago but less than 5 years ago
   - 5 or more years ago
   - Never received dental care
2. What was the reason of your last visit to the dentist?

(You can choose more than one option)

- - Consultation/advise
  - Pain or trouble with teeth, gums or mouth
  - Treatment/ follow-up treatment
  - Routine check-up
  - Don’t know/don’t remember
  - Never received dental care

1. How long is it since you last saw a dentist in Germany?
   - Less than 1 month ago
   - 1–2 months ago
   - 3–5 months ago
   - 6 or more months ago
   - I did not visit a dentist in Germany
2. What was the reason of your last visit to the dentist in Germany?

(You can choose more than one option)

- - Consultation/advise
  - Pain or trouble with teeth, gums or mouth
  - Treatment/ follow-up treatment
  - Routine check-up
  - Don’t know/don’t remember
  - I did not visit a dentist in Germany

1. How often did you normally go to the dentist in Ukraine?
   - Once a year
   - Twice a year
   - Four or more times a year
   - Less than once a year
   - Never received dental care
   - Don’t know/don’t remember
2. Because of the state of your teeth or mouth, how often have you experienced any of the following problems during the past 12 months?

(Read each item)

|  | **Very often** | **Fairly often** | **Sometimes** | **Never** | **Don’t know** |
| --- | --- | --- | --- | --- | --- |
| Difficulty in biting foods | 🞏 | 🞏 | 🞏 | 🞏 | 🞏 |
| Difficulty chewing foods | 🞏 | 🞏 | 🞏 | 🞏 | 🞏 |
| Difficulty with speech/trouble pronouncing words | 🞏 | 🞏 | 🞏 | 🞏 | 🞏 |
| Dry mouth | 🞏 | 🞏 | 🞏 | 🞏 | 🞏 |
| Felt embarrassed due to appearance of teeth | 🞏 | 🞏 | 🞏 | 🞏 | 🞏 |
| Felt tense because of problems with teeth or mouth | 🞏 | 🞏 | 🞏 | 🞏 | 🞏 |
| Have avoided smiling because of teeth | 🞏 | 🞏 | 🞏 | 🞏 | 🞏 |
| Had sleep that is often interrupted | 🞏 | 🞏 | 🞏 | 🞏 | 🞏 |
| Have taken days off work | 🞏 | 🞏 | 🞏 | 🞏 | 🞏 |
| Difficulty doing usual activities | 🞏 | 🞏 | 🞏 | 🞏 | 🞏 |
| Felt less tolerant of spouse or people who are close to you | 🞏 | 🞏 | 🞏 | 🞏 | 🞏 |
| Have reduced participation in social activities | 🞏 | 🞏 | 🞏 | 🞏 | 🞏 |

1. How often do you eat or drink any of the following foods, even in small quantities?

(Read each item)

|  | **Several times a day** | **Every day** | **Several times a week** | **Once a week** | **Several times a month** | **Seldom/never** |
| --- | --- | --- | --- | --- | --- | --- |
| Fresh fruit | 🞏 | 🞏 | 🞏 | 🞏 | 🞏 | 🞏 |
| Biscuits, cakes, cream cakes | 🞏 | 🞏 | 🞏 | 🞏 | 🞏 | 🞏 |
| Sweet pies, buns | 🞏 | 🞏 | 🞏 | 🞏 | 🞏 | 🞏 |
| Jam or honey | 🞏 | 🞏 | 🞏 | 🞏 | 🞏 | 🞏 |
| Chewing gum containing sugar | 🞏 | 🞏 | 🞏 | 🞏 | 🞏 | 🞏 |
| Sweets/candy | 🞏 | 🞏 | 🞏 | 🞏 | 🞏 | 🞏 |
| Lemonade, Coca Cola or other soft drinks | 🞏 | 🞏 | 🞏 | 🞏 | 🞏 | 🞏 |
| Tea with sugar | 🞏 | 🞏 | 🞏 | 🞏 | 🞏 | 🞏 |
| Coffee with sugar | 🞏 | 🞏 | 🞏 | 🞏 | 🞏 | 🞏 |

1. How often do you use any of the following types of tobacco?

(Read each item)

|  | **Every day** | **Several times a week** | **Once a week** | **Several times a month** | **Seldom** | **Never** |
| --- | --- | --- | --- | --- | --- | --- |
| Cigarettes | 🞏 | 🞏 | 🞏 | 🞏 | 🞏 | 🞏 |
| Cigars | 🞏 | 🞏 | 🞏 | 🞏 | 🞏 | 🞏 |
| A pipe | 🞏 | 🞏 | 🞏 | 🞏 | 🞏 | 🞏 |
| Chewing tobacco | 🞏 | 🞏 | 🞏 | 🞏 | 🞏 | 🞏 |
| Use snuff | 🞏 | 🞏 | 🞏 | 🞏 | 🞏 | 🞏 |
| Other | 🞏 | 🞏 | 🞏 | 🞏 | 🞏 | 🞏 |
| Please specify____________________________________________________________________ | | | | | | |

1. During the past 30 days, on the days you drank alcohol, how many drinks did you usually drink per day?
   - Less than 1 drink
   - 1 drink
   - 2 drinks
   - 3 drinks
   - 4 drinks
   - 5 or more drinks
   - Did not drink alcohol during the past 30 days

**Dental care access**

1. Based on your experience, how much do these factors in your opinion limit your access to dentist in Germany?

|  | **Strongly disagree** | **Disagree** | **Somewhat disagree** | **Somewhat agree** | **Agree** | **Strongly agree** |
| --- | --- | --- | --- | --- | --- | --- |
| **Language barriers, such as**  - I don’t understand the language  - I cannot explain what my problem is | 🞏 | 🞏 | 🞏 | 🞏 | 🞏 | 🞏 |
| **Financial barriers, such as**  - I am afraid that I need to pay too much  - I could not financially afford to | 🞏 | 🞏 | 🞏 | 🞏 | 🞏 | 🞏 |
| **Transport barriers**  **-** It was not possible for me to physically reach the facility | 🞏 | 🞏 | 🞏 | 🞏 | 🞏 | 🞏 |
| **Availability in my region** | 🞏 | 🞏 | 🞏 | 🞏 | 🞏 | 🞏 |
| **Complicated health care system**  - I don’t understand how the (dental) health care system works in Germany | 🞏 | 🞏 | 🞏 | 🞏 | 🞏 | 🞏 |
| **Problems with finding a dentist**  - I don’t know how to find and register with a dentist  - I don’t know a good dentist | 🞏 | 🞏 | 🞏 | 🞏 | 🞏 | 🞏 |
| **Dental anxiety**  - I am afraid of the treatment or the dentist | 🞏 | 🞏 | 🞏 | 🞏 | 🞏 | 🞏 |
| **Trust issues**  - I don’t trust the dentists here in Germany | 🞏 | 🞏 | 🞏 | 🞏 | 🞏 | 🞏 |
| **Cultural and religious beliefs**  - I don’t go to the dentist because of my culture or religion | 🞏 | 🞏 | 🞏 | 🞏 | 🞏 | 🞏 |
| **Social/domestic barriers**  - Treatment/consultation is not possible for me because of my schedule  - Someone needs to stay with my children | 🞏 | 🞏 | 🞏 | 🞏 | 🞏 | 🞏 |

**Unmet needs for dental health services**

- 1. Was there any time in Germany when you needed to consult a dentist but did not?
     - Yes
     - No
     - No need for examination or treatment
  2. If you answered previous question with “Yes”: What was the reason?

|  | **Yes** | **No** |
| --- | --- | --- |
| **Language barriers**  - I don’t understand the language  - I cannot explain what my problem is | 🞏 | 🞏 |
| **Financial barriers**  - I am afraid that I need to pay too much  - I could not financially afford to | 🞏 | 🞏 |
| **Transport barriers**  **-** It was not possible for me to physically reach the facility | 🞏 | 🞏 |
| **Availability in my region** | 🞏 | 🞏 |
| **Complicated health care system**  - I don’t understand how the (dental) health care system works in Germany | 🞏 | 🞏 |
| **Problems with finding a dentist**  - I don’t know how to find and register with a dentist  - I don’t know a good dentist | 🞏 | 🞏 |
| **Dental anxiety**  - I am afraid of the treatment or the dentist | 🞏 | 🞏 |
| **Trust issues**  - I don’t trust the dentists here in Germany | 🞏 | 🞏 |
| **Cultural and religious beliefs**  - I don’t go to the dentist because of my culture or religion | 🞏 | 🞏 |
| **Social/domestic barriers**  - Treatment/consultation is not possible for me because of my schedule  - Someone needs to stay with my children | 🞏 | 🞏 |
| **Other** | 🞏 | 🞏 |
| Please specify______________________________________________________________ | | |

- 1. Did you start a dental treatment in Ukraine, that needs to be continued in Germany?

*(for example, orthodontic treatment)*

- - - Yes
    - No
    - I don’t know
  1. If you answered previous question with “Yes”: Could you find a dentist and continue this treatment in Germany?
     - Yes
     - No
     - I did not search a dentist

1. How do you rate the quality of dental care in Germany in general?
   - Very good
   - Good
   - Average
   - Poor
   - Very poor
   - Don’t know

**Stress measurement**

In this final part of the survey, you'll answer a few questions about your feelings and emotions.

1. Over the last 2 weeks, how often have you been bothered by any of the following problems?

|  | | | **Not at all** | **Several days** | | **More than half the days** | **Nearly every day** |
| --- | --- | --- | --- | --- | --- | --- | --- |
| **1.** Little interest or pleasure in doing things | | | 🞏 | 🞏 | | 🞏 | 🞏 |
| **2.** Feeling down, depressed, or hopeless | | | 🞏 | 🞏 | | 🞏 | 🞏 |
| **3.** Trouble falling or staying asleep, or sleeping too much | | | 🞏 | 🞏 | | 🞏 | 🞏 |
| **4.** Feeling tired or having little energy | | | 🞏 | 🞏 | | 🞏 | 🞏 |
| **5.** Poor appetite or overeating | | | 🞏 | 🞏 | | 🞏 | 🞏 |
| **6.** Feeling bad about yourself — or that you are a failure or  have let yourself or your family down | | | 🞏 | 🞏 | | 🞏 | 🞏 |
| **7.** Trouble concentrating on things, such as reading the newspaper or watching television | | | 🞏 | 🞏 | | 🞏 | 🞏 |
| **8.** Moving or speaking so slowly that other people could have noticed? Or the opposite — being so fidgety or restless that you have been moving around a lot more than usual | | | 🞏 | 🞏 | | 🞏 | 🞏 |
| **9.** Thoughts that you would be better off dead or of hurting yourself in some way | | | 🞏 | 🞏 | | 🞏 | 🞏 |
| **If you checked off any problems, how difficult have these problems made it for you to do your work, take care of things at home, or get along with other people?** | | | | | | | |
| **Not difficult at all**  🞏 | **Somewhat difficult**  🞏 | **Very difficult**  🞏 | | | **Extremely difficult**  🞏 | | |

**Anxiety measurement**

1. Over the last 2 weeks, how often have you been bothered by any of the following problems?

|  | **Not at all** | **Several days** | **More than half the days** | **Nearly every day** |
| --- | --- | --- | --- | --- |
| **1.** Feeling nervous, anxious or on edge | 🞏 | 🞏 | 🞏 | 🞏 |
| **2.** Not being able to stop or control worrying | 🞏 | 🞏 | 🞏 | 🞏 |
| **3.** Worrying too much about different things | 🞏 | 🞏 | 🞏 | 🞏 |
| **4.** Trouble relaxing | 🞏 | 🞏 | 🞏 | 🞏 |
| **5.** Being so restless that it is hard to sit still | 🞏 | 🞏 | 🞏 | 🞏 |
| **6.** Becoming easily annoyed or irritable | 🞏 | 🞏 | 🞏 | 🞏 |
| **7.** Feeling afraid as if something awful might happen | 🞏 | 🞏 | 🞏 | 🞏 |

**Thank you for participating in this study!**

**Please send the questionnaire within two weeks back to:**

E-Mail: [maksym.ponomarenko@rwth-aachen.de](mailto:maksym.ponomarenko@rwth-aachen.de)

Postal address:

PD Dr. med. Andrea Kaifie-Pechmann and Maksym Ponomarenko

Institut für Arbeits-, Sozial- und Umweltmedizin

Uniklinikum RWTH Aachen

Pauwelsstraße 30 52074 Aachen

Deutschland

**If you want to participate in a prize draw leave your e-mail address below:**

**E-mail: ___________________________________________________________**
