# Supplementary material for: Oral health, stress and barriers accessing dental health care among war-affected Ukrainian refugees in Germany
Source: BMC Oral Health. 2023 Oct 27;23:804. doi: 10.1186/s12903-023-03513-x (PMC10612176; doi:10.1186/s12903-023-03513-x)
Supplement: Supplementary file 2 — Supplementary Material 2 [file 12903_2023_3513_MOESM2_ESM.doc]

**Supplementary Table 1: Barriers to access dental health care**

|  | **Financial barriers** | **Language barriers** | **Complicated health care system** | **Problems with finding a dentist** | **Trust issues** | **Dental anxiety** | **Social / domestic barriers** | **Failed a consultation** |
| --- | --- | --- | --- | --- | --- | --- | --- | --- |
| **Gender (n (%))** | | | | | | | | |
| Males | 52 (80) | 53 (81.5) | 53 (81.5) | 47 (72.3) | 14 (21.9)* | 19 (29.2) | 11 (17.2) | 34 (53.1) |
| Females | 482 (82.7) | 478 (82.3) | 428 (73.5) | 414 (71.1) | 230 (39.6)* | 207 (35.6) | 139 (24.1) | 352 (60.3) |
| **Age (mean (SD))** | | | | | | | | |
| Agreed | 37.6 (10.7) | 37.8 (10.6)* | 37.3 (10.4) | 37.3 (10.7) | 35.1 (9.0)** | 36.2 (10.6)* | 33.9 (8.3)** | 37.02 (9.64) |
| Did not agree | 36.9 (10.6) | 35.3 (10.5)* | 37.8 (11.4) | 37.7 (10.7) | 38.8 (11.4)** | 38.1 (10.7)* | 38.4 (11.0)** | 37.90 (11.78) |
| **Education** | | | | | | | | |
| Higher Education |  |  |  |  |  |  |  | 278 (58.8) |
| Other |  |  |  |  |  |  |  | 110 (61.8) |
| **Size of the city in Germany (n (%))** | | | | | | | | |
| Large or medium | 450 (81.8) | 445 (81.2) | 411 (74.9) | 399 (72.4) | 218 (39.8)* | 185 (33.6%) | 128 (23.5) |  |
| Small or village | 89 (86.4) | 90 (87.4) | 73 (70.9) | 66 (65.4) | 28 (27.5)* | 43 (42.6%) | 25 (24.5) |  |
| **Time in Germany (n (%))** | | | | | | | | |
| Less than 4 months | 416 (89.2)* | 119 (86.2) | 107 (77.5) | 102 (73.8) | 44 (32.1) | 51 (36.7) | 25 (24.5) |  |
| 4 months or more | 89 (80.9)* | 416 (81.1) | 377 (73.4) | 363 (70.8) | 203 (39.6) | 178 (34.7) | 128 (23.5) |  |
| **Arrived alone or with family (n (%))** | | | | | | | | |
| Alone | 101 (88.6) | 89 (78.8) | 79 (69.9) | 86 (75.4) | 39 (34.8) | 38 (33.3) | 14 (12.6)* | 55 (48.3)* |
| With family | 439 (81.3) | 447 (82.9) | 405 (75.0) | 380 (70.5) | 208 (38.6) | 191 (35.4) | 139 (25.9)* | 335 (62.0)* |
| **State of teeth (n (%))** | | | | | | | | |
| Poor / very poor |  |  |  |  |  |  |  | 111 (74.5)** |
| Average or better |  |  |  |  |  |  |  | 337 (58.1)** |
| **State of gums (n (%))** | | | | | | | | |
| Poor / very poor |  |  |  |  |  |  |  | 44 (75.9)* |
| Average or better |  |  |  |  |  |  |  | 337 (58.1)* |
| *Multiple choice was available*  *** - p ≤ .0001; * - p < .05* | | | | | | | |  |
